# Ultrasounds could be considered as a future tool for probing growing bone properties

**DOI:** 10.1038/s41598-020-72776-z

**Published:** 2020-09-24

**Authors:** Emmanuelle Lefevre, Cécile Baron, Evelyne Gineyts, Yohann Bala, Hakim Gharbi, Jean-Marc Allain, Philippe Lasaygues, Martine Pithioux, Hélène Follet

**Affiliations:** 1 Aix Marseille Univ, CNRS,ISM, Marseille, France; 2 Aix Marseille Univ, APHM,CNRS, ISM, Sainte-Marguerite Hospital, Institute for Locomotion, Department of Orthopaedics and Traumatology, Marseille, France; 3 Univ Lyon, Univ Claude Bernard Lyon 1, INSERM, LYOS UMR1033, F69008 Lyon, France; 4 LMS, Ecole Polytechnique,CNRS, Institut Polytechnique de Paris, Palaiseau, France; 5 Inria, Palaiseau, France; 6 Laboratoire Vibrations Acoustique, INSA Lyon, Campus LyonTech la Doua, Villeurbanne, France; 7 Aix Marseille Univ., CNRS, Centrale Marseille, LMA, Marseille, France

**Keywords:** Ageing, Bone development, Mechanical engineering, Mechanical properties

## Abstract

Juvenile bone growth is well described (physiological and anatomical) but there are still lacks of knowledge on intrinsic material properties. Our group has already published, on different samples, several studies on the assessment of intrinsic material properties of juvenile bone compared to material properties of adult bone. The purpose of this study was finally to combine different experimental modalities available (ultrasonic measurement, micro-Computed Tomography analysis, mechanical compression tests and biochemical measurements) applied on small cubic bone samples in order to gain insight into the multiparametric evaluation of bone quality. Differences were found between juvenile and adult groups in term of architectural parameters (Porosity Separation), Tissue Mineral Density (TMD), diagonal stiffness coefficients (C_33_, C_44,_ C_55,_ C_66_) and ratio between immature and mature cross-links (CX). Diagonal stiffness coefficients are more representative of the microstructural and biochemical parameters of child bone than of adult bone. We also found that compression modulus E was highly correlated with several microstructure parameters and CX in children group while it was not at all correlated in the adult group. Similar results were found for the CX which was linked to several microstructure parameters (TMD and E) only in the juvenile group. To our knowledge, this is the first time that, on a same sample, ultrasonic measurements have been combined with the assessment of mechanical and biochemical properties. It appears that ultrasonic measurements can provide relevant indicators of child bone quality (microstructural and biochemical parameters) which is promising for clinical application since, B-mode ultrasound is the preferred first-line modality over other more constraining imaging modalities (radiation, parent–child accessibility and access to the patient's bed) for pediatric patients.

## Introduction

The need for a better clinical understanding of juvenile bone is essential to improve the diagnosis of congenital or acquired diseases and trauma that may influence bone development. Even though bone growth is well described^1^, there are still lacks of knowledge on intrinsic material properties. Compositional parameters, geometry, material properties, architecture and microstructure are associated with bone fragility and remains difficult to evaluate^2^.

Collagen is the main organic component of bone (80%). Collagen fibers play an essential role in human cortical bone mechanical integrity^3–8^. as it provides ductility to bone whereas the mineral phase provides rigidity^5, 8^. It is well established that part of the changes in bone mechanical properties with aging are related to changes in collagen^9, 10^.

In bone tissue, two different collagen cross-linking processes can be outlined: immature and matures cross-links. Immature cross-links are di-valent crosslinks (DHLNL and HLNL) that become tri-valent mature cross-link after the formation of pyridinoline (PYD) and deoxypyridinoline (DPD). The ratio between immature and mature cross-links is a determinant of bone quality and is called the CX ratio in the current study^9, 11^. This ratio should decrease with age. Our group has already published, on different sets of samples (as femurs, ribs or fibulas), several studies on the investigation of intrinsic tissue properties of juvenile bone compared to material properties of adult bone. Berteau et al.^9^ have shown that enzymatic cross-links ratio affect the post-yield cortical bone behaviour.

The elastic properties of adult bone ex vivo can be assessed by ultrasonic methods^12^. By measuring both shear and compression ultrasonic bulk wave velocities on a single specimen to calculate the stiffness coefficients^13–18^. We have recently published the values of the diagonal coefficients of the stiffness tensor measured on children bone specimens, gaining insight into how the anisotropy is age-related^19^.

To complete these analyses, the morphometry of the vascular porosity network of these samples was analysed, showing that the microstructural variations of the pore network contribute to modify the mechanical behaviour in compression and shear evaluated by ultrasonic methods^20^. At the tissue level^21^, the micro-indentation allowed the assessment of the mechanical properties of the extracellular matrix. The Fourier Transform Infrared Microspectroscopy (FTIRM) is used to assess the physicochemical modifications of bone composition (organic versus mineral matrix). Quantitative microradiography measurement provided the degree of bone mineralization (DMB). In this work, it was clearly established that child bone has a different mechanical behavior from that of adult bone^21^. Nevertheless, the characterization of juvenile bone remains poorly documented despite some recent studies on the subject^22^.

The main challenge remains to evaluate, on the same sample, the different characteristics that determine bone quality in children as the different experimental modalities needed for this multi-parametric characterization are rarely available at the same time (example with the FTIRM and quantitative mineralization in^21^).

The primary motivation of this study is to combine the different experimental modalities available (Ultrasounds measurements, micro-CT analysis, mechanical compression tests and biochemical measurements) applied on the same small cubic cortical bone samples from adult and juvenile groups. It contributes to the ultimate goal of evaluating whether there is a clinical potential for ultrasounds to measure bone properties in different populations.

## Material and methods

### Specimens

As previously published^21^, bone samples were collected at the same location from the distal third of the fibula of 13 children (8 male and 5 female) 6–17 years old (mean age of 12 years ± 3 years) during corrective surgery for a growth plate fracture, clubfeet, or for chondrodystrophy, hypoplasia, epiphyseal dysplasia. Surgeries were performed at the Timone Hospital (Marseille, France). All children were ambulatory prior to surgery and none of them received medications known to affect bone remodelling. In accordance with the French Code of Public Health, the National Commission for Data Protection and Liberties (CNIL-France) accepted the experimental protocol and ethical approval was granted by the anatomy laboratory.

Adult bone samples were collected from the same anatomical location as the children bone samples (the distal third of the fibula) from 16 donors (7 male and 9 female) 50–95 years old (mean age of 75 years ± 13 years). Autopsies were performed to build a bone sample bank (French body donation to science program, accepted the experimental protocol and ethical approval, declaration number: DC-2015-2357; Laboratory of Anatomy, Faculty of Medicine Lyon Est, University of Lyon, France). Before cut, all samples were maintained frozen at − 20 °C wrapped in Phosphate Buffer Saline solution-soaked gauze.

### Ethical approval

All procedures performed in this study were approved by: The National Consultative Ethics Committee for Health and Life Sciences and The National Commission for Data Protection and Liberties (CNIL-France) for juveniles samples, and French body donation to science program for adults donors (Approval Number: DC-2015-2357).

### Informed consent

Informed consent was obtained from the children’s legal representatives. Informed consent for donors was obtained from individual before their death by the French body donation to science program.

#### Nota

Samples are slightly different than those previously studied in^19, 20^, and totally different than the ones used in^21^. Moreover, ultrasounds and biochemistry were also obtained from 8 other juveniles’ femurs. But, for those samples, there are not enough data for statistical analysis, raw data are given in supplementary material as open source data and were not included into this study (supplementary Table 1).

### Cubic sample preparation

**Figure 1:**
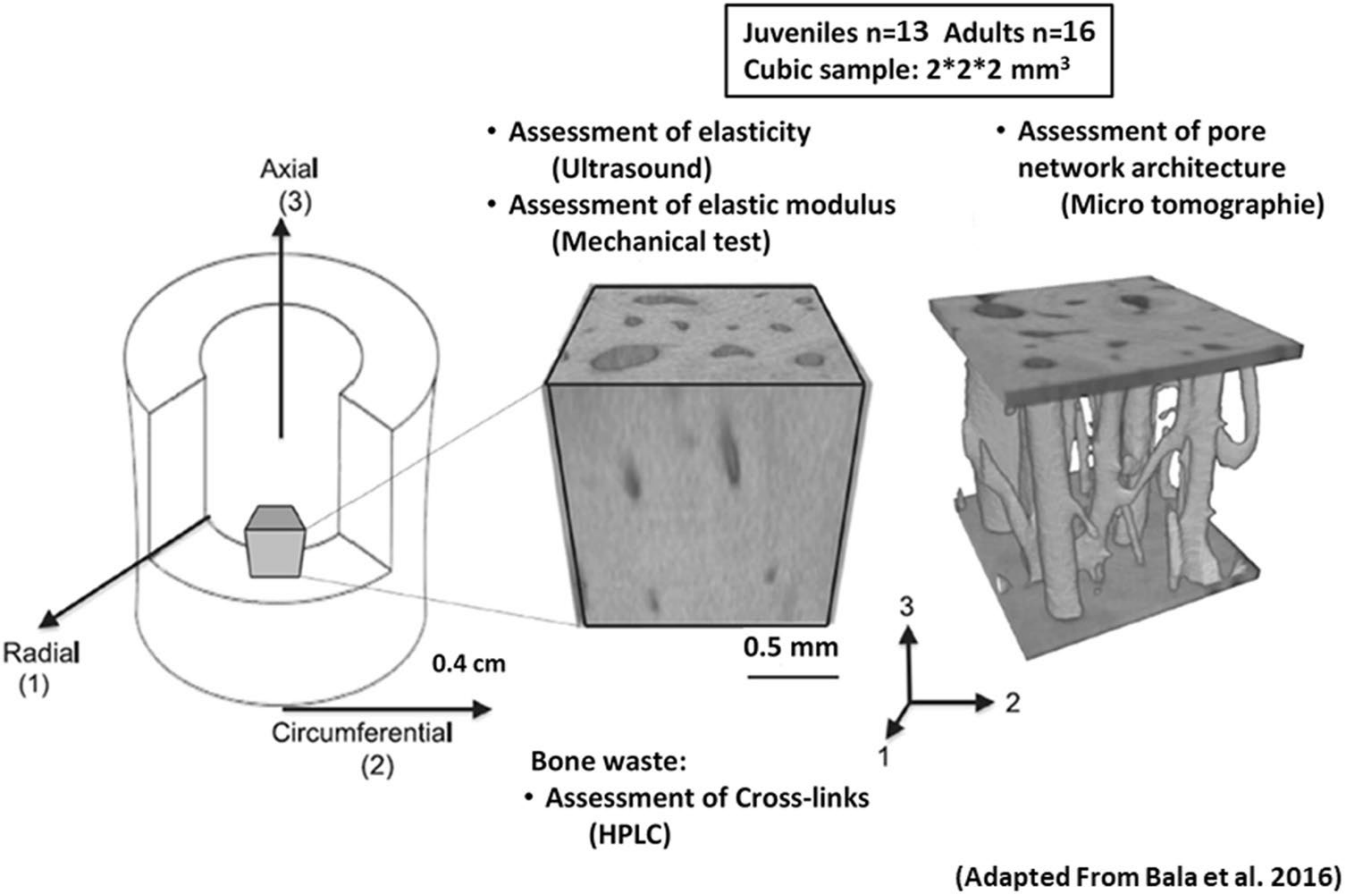
From left to Right, bone cubic samples were cored from the distal fibula and oriented according to the radial (1), circumferential (2) and axial (3) axes for assessment of the elastic coefficients by ultrasounds and pore network architecture using micro-CT. Cross-links measurement was done using the surrounding bone. Reprinted from Bala et al.^20^, Copyright (2019), with permission from Elsevier.

As previously described, samples were slowly thawed and then cut with a water-cooled low-speed diamond saw (Buehler Isomet 4000, Buehler, Lake Bluff, IL, USA) into cubic parallelepipeds (dimensions: 2 × 2 × 2mm^3^; mean = 1.96 ± 0.56 mm). The faces of the specimens were oriented according to the radial (axis 1), tangential (axis 2) and axial (axis 3) directions defined by the anatomic shape of the bone diaphysis, as previously published^19, 20^ (Fig. 1). Once cut, the cubic samples were maintained frozen at − 20 °C wrapped in Phosphate Buffer Saline solution-soaked gauze.

### Ultrasounds measurement

Stiffness coefficients were evaluated via the ultrasonic measurement protocol described in^19^. Shortly, two types of measurements were implemented: compression wave velocity measurement and shear wave velocity measurement. For the first one, the cubic bone sample was placed over a gelatin block (agar) to keep it aligned between two transducers (VP1093, 1 MHz, CTS Valpey Corporation, Hopkinton, MA) immersed in water. The difference between the propagation times with and without sample (i.e. in water only) was measured and the compression wave velocity calculated. The second type of measurements was made with two transverse wave transducers (Panametrics V156, 5 MHz, Inc., Waltham, MA) in contact with the bone sample. A reference measurement of the time of flight of the shear wave was made in a 5 mm-thick aluminum sample, and the delay between this reference time and the time of flight through the bone sample was estimated to calculate the shear wave velocity. The two types of measurement were done in the three directions of the cubic bone sample (radial, tangential and axial) allowing to assess the 6 diagonal stiffness coefficients of C_ii_ (i = 1.0.6) of each bone sample after measuring the mass density with a micrometric balance equipped with a density kit (Voyager 610, Ohaus Corporation, FlorhamPark, NJ, USA, measurement uncertainty of 0.001 g/cm^3^).

### Micro CT analysis

The samples were analysed following the same methods as in^20^. Briefly, the cubic bone samples were imaged using a desktop micro-CT system (Skyscan1174, Bruker, Kontich, Belgium). Scanning was done with the bone specimen immerged in distilled water in a 6 mm inner diameter plastic tube and held in place thanks to gauze. Sample axial axis (i.e., Haversian canals' principal orientation) was aligned to the rotation axis of the sample holder. As recommended by^23^, scans were performed with a field of view of 1024 × 1024 pixels, a source voltage 50 kV, current 800 mA, rotation step 0.6° over a 360° rotation and a 0.5 mm-thick aluminum filter for beam hardening reduction. An isotropic voxel size of 8.14 µm was used with an exposure time of 4 s, 2 frames averaging leading to a total scan time of 82 min for each sample. Images were reconstructed using a filtered back-projection algorithm (NRecon software, V1.6.9, SkyscanNV, Kontich, Belgium). For each cube, a stack of 210 sections was reconstructed. Within the tissue volume (TV, mm^3^), the porosity (void volumes) was segmented as a solid and the mineralized bone as a background by using global thresholding^20^.

The following morphometric variables were calculated using (CTAn software, V1.16.4.1 (64bits) SkyscanNV, Kontich, Belgium): the pore volume fraction (Po.V/TV, with Po.V = Pore Volume and TV: tissue volume, %), the pore surface to pore volume ratio (Po.S/Po.V, 1/mm). Pore diameter (Po.Dm, mm) corresponding to the average diameter of the pores and pore separation (Po.Sp, mm) corresponding to the average separation between pores were both calculated using a sphere-fitting algorithm. As an index of heterogeneity in the intra-individual distribution of pore size and separation, the standard deviation of Po.Dm and Po.Sp are reported as Po.Dm.SD and Po.Sp.SD both expressed in mm. The pore number (Po.N, 1/mm) was calculated as: Po.N = 1/(Po.Sp + Po.Dm). Connectivity (Conn.Density, 1/mm^3^) was evaluated by the Euler characteristic according to the method detailed by^24^ and normalized by the TV. We also assessed the pore pattern factor (Po.Pf in analogy with the trabecular pattern factor, 1/mm) calculated as the ratio of volumes and surfaces before and after dilation. Lower Po.Pf indicates higher concavity i.e. better-connected pore network. Structure Model Index (SMI, no unit^25^,) and Degree of Anisotropy (DA, no unit^24^) are also reported. For reminder, in trabecular bone, SMI quantify the type of trabecular bone: plate (SMI = 0) and rod (SMI = 3), and DA quantify the anisotropy of the structure (from 0: total isotropy, to 1: total anisotropy). Tissue mineral density was also calculated (g/cm^3^) from the grey level and calibration phantom^20^.

### Compression test on cortical bone cube

**Figure 2:**
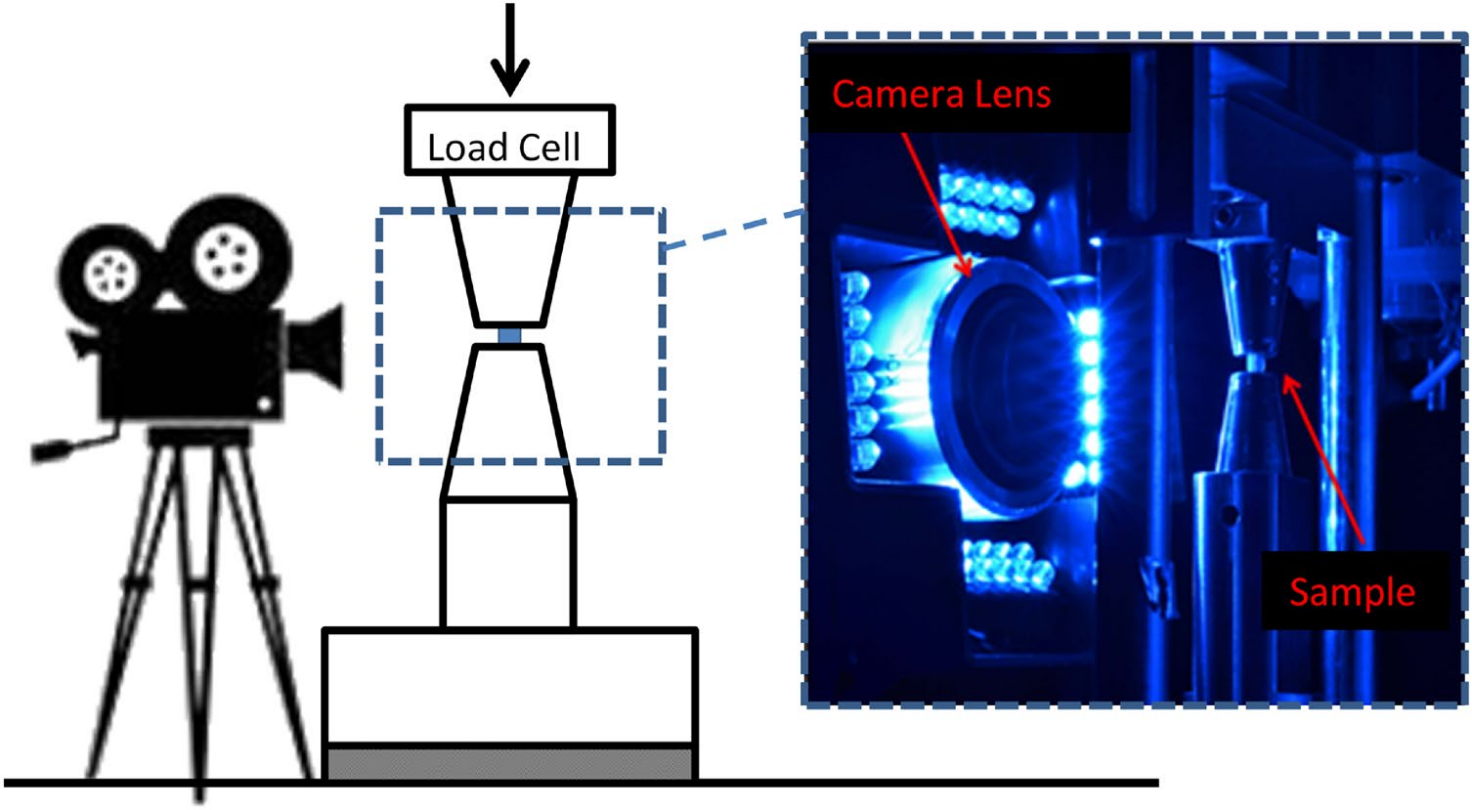
Schematic mechanical compressive test device. High resolution camera records the cubic sample motion under compression.

Cortical cubic bones samples (2 * 2 * 2 mm^3^) were mechanically loaded in a compression test configuration, with a quasi-static loading (load speed: 1 µm s^−1^, strain rate around 10^–5^ s^−1^ neglecting machine compliance, Fig. 2) applied in the axial direction of the samples. They were kept wet during the test by adding physiological saline solution. A speckle was painted on the specimen to compute 2D surface strain field. Image acquisition was done by a CCD camera (Pike-Allied Vision Technologies Prosilica GX, Surrey, UK) with an Edmund Optics lens (× 10 Mitutoyo, Japan), combined with an autofocus system. Images are cropped from the non-relevant parts into 2452 × 2452 pixels images, with square pixels of 0.6 µm. The mean strain on the field of view was then extracted using custom software (Silvia), giving the true strain. Resolution of the strain measurement was estimated to be less than 10^–2^%^26^. Images were acquired with a rate around 1 image every 20 s (depending on the autofocus time). At the same time, the force was measured through a 5000 N load cell (Futek, USA) every 1 s (as well as the displacement of the compression machine). Stress was obtained by dividing the measured force by the compressed surface of the sample. Machine strain has to be corrected from the machine compliance, while the optical strain is the true one. However, the machine strain is obtained every second, while the true one is obtained at a slower rate. For statistical comparison between juvenile and adult bone properties, we extracted the compression modulus, the Poisson’s ratio and the maximal stress (using the optical measure for the true strain).

**Table 1:** Descriptive statistics. Difference between juvenile/adult is shown using a Mann–Whitney unpaired test.

	Juveniles (n = 13) (6–17 years, 12 ± 3) Mean (SD)	Adults (n = 16) (50–95 years, 75 ± 13) Mean (SD)	Mann–Whitney *p* value
**Microstructure**			
Po.V/TV (%)	13.0 (9.76)	16.11 (10.16)	0.30
Po.S/Po.V (1/mm)	37.8 (14.3)	36.9 (13.8)	0.8358
Po.N (1/mm)	0.76 (0.41)	0.91 (0.35)	0.1742
Po.Dm (µm)	159 (62)	161 (81)	0.8719
Po.Dm.SD (µm)	77 (40)	79 (51)	0.7297
Po.Sp (µm)	383 (56)	317 (57)	**0.0106**
Po.Sp.SD (µm)	122 (17)	101 (21)	**0.0092**
Conn.Density (1/mm^3^)	7.9 (6.0)	22.5 (15.7)	**0.0030**
DA	0.739 (0.05)	0.729 (0.078)	0.8836
Po.Pf	0.021 (0.007)	0.017 (0.006)	0.1243
SMI	3.32 (0.71)	2.96 (0.31)	0.0621
TMD (g/cm^3^)	1.020 (0.085)	1.096 (0.028)	**0.0034**
**Elastic coefficient (GPa)**			
C11	15.8 (3.4)	18.12 (4.07)	0.0956
C22	15.5 (3.4)	18.37 (6.14)	0.0655
C33	22.9 (5.1)	28.51 (5.07)	**0.0124**
C44	4.02 (0.79)	4.79 (0.70)	**0.0226**
C55	3.91 (0.81)	4.94 (1.05)	**0.0075**
C66	3.00 (0.44)	3.64 (9.6)	**0.0097**
**Biochemistry (mmol/mol collagen)**			
DPD	99 (23)	128 (22)	**0.0050**
PYD	409 (131)	497 (85)	0.0722
DHLNL	1891 (637)	424 (120)	** < 0.0001**
HLNL	625 (229)	122 (35)	** < 0.0001**
[DHLNL + HLNL]	2517 (836)	546 (148)	** < 0.0001**
[PYD + DPD]	508 (151)	626 (99)	**0.0485**
[PYD/DPD]	4.040 (0.93)	3.9 (0.6)	0.4299
CX = [DHLNL + HLNL]/ [PYD + DPD]	5.09 (1.63)	0.88 (0.20)	** < 0.0001**
**Compression parameters**			
	(Nota: n = 11)	(Nota: n = 12)	
Maximal stress (MPa)	203 (77)	160 (40)	0.0648
Elastic modulus (GPa)	12.2 (5.5)	10.2 (3.5)	0.2954
Poisson’s coefficient	0.446 (0.12)	0.46 (0.15)	0.8535

Bold correspond to significant difference.

### Biochemical measurements

The cubic bone samples used for mechanical (ultrasounds and compression) experiments were not used for biochemical measurements in order to keep them intact for further analysis using other modalities. In that way, cortical bone overages from the previously enounced sample preparation were kept frozen at − 80 °C for the biochemical measurements. This means that these measurements were done on a tissue closely surrounding the region previously used for the mechanical experiments. The availability of matter, the final database for biochemical measurements is composed of 29 cortical samples (16 adults and 13 juveniles).

Detailed protocols of biochemistry analysis have been already published^11^. Briefly, samples were cut into small pieces, powered in liquid nitrogen and demineralized with an EDTA solution. The samples were then reduced in NaBH_4_ to stabilized immature crosslinks DHLNL and HLNL, and then hydrolysed in hydrochloric acid. PYD and DPD mature cross-links and DHLNL and HLNL immature cross-links were extracted and concentrated from the hydrolysates using a Bond Elut Cellulose solid phase extraction column (Agilent Technologies, Santa Clara, CA, USA). Then cross-links were separated on a reversed phase C18 column and quantified by mass spectrometry using an Alliance 2695 separation HPLC system and ZQ Mass detector (Waters Corp. Milford, MA, USA). The total amount of collagen was determined by hydroxyproline HPLC assay (Biorad, München, Germany). Crosslink concentrations are given in mmol by mol of collagen^11, 27, 28^. The ratio CX = [DHLNL + HLNL] / [PYD + DPD] was used to express the state of cross-links maturation, which assessed collagen matrix maturation^9, 11^.

### Statistical analysis

Twenty-nine samples were tested and values for multiple samples from the same donor were averaged, yielding one value per donor (juveniles n = 13, adults n = 16). We did not test mechanically all the samples which went through both microstructural and CX measurements.

Statistical analysis was performed in SPSS 20.0 (IBM, Amonk, NY, USA) using a significance level of 5%. In Table 2, significance between 0.051 < *p* < 0.099 were indicated as “Borderline (bl)”. Data are reported as mean ± SD unless otherwise stated. All tests were two-tailed. Due to the small number of samples into each group, non-parametric tests were used. The Mann–Whitney unpaired test was used to test for differences between juvenile and adult groups. The influence of the microstructure on mechanical behaviour was studied using bivariate correlations that were tested by non-parametric Spearman’s rank correlation test.

## Results

We analysed the effect of gender using a multiple regression on adult and juvenile bone, and no influence of sex was found.

Scatterplots depending on age are shown in Fig. 4A–H.

### Comparison juveniles versus adults

Table 1 shows the descriptive statistics and difference between juvenile and adult groups for microstructure, mechanics (ultrasounds and compression) and biochemistry parameters.

In term of microstructure, results were similar to those previously published^20^, with a higher pore number (Po.N) and pore volume fraction (Po.V/TV) in adult bone samples compared to juvenile ones (not reached significance level, *resp.* + 20% and + 24%). The higher connectivity (Conn.Density) in adult samples (+ 185%) was not reflected by the degree of anisotropy (DA) which is identical in the two groups. Tissue mineral density (TMD) was significantly higher in adults group compared to juveniles one.

**Figure 3:**
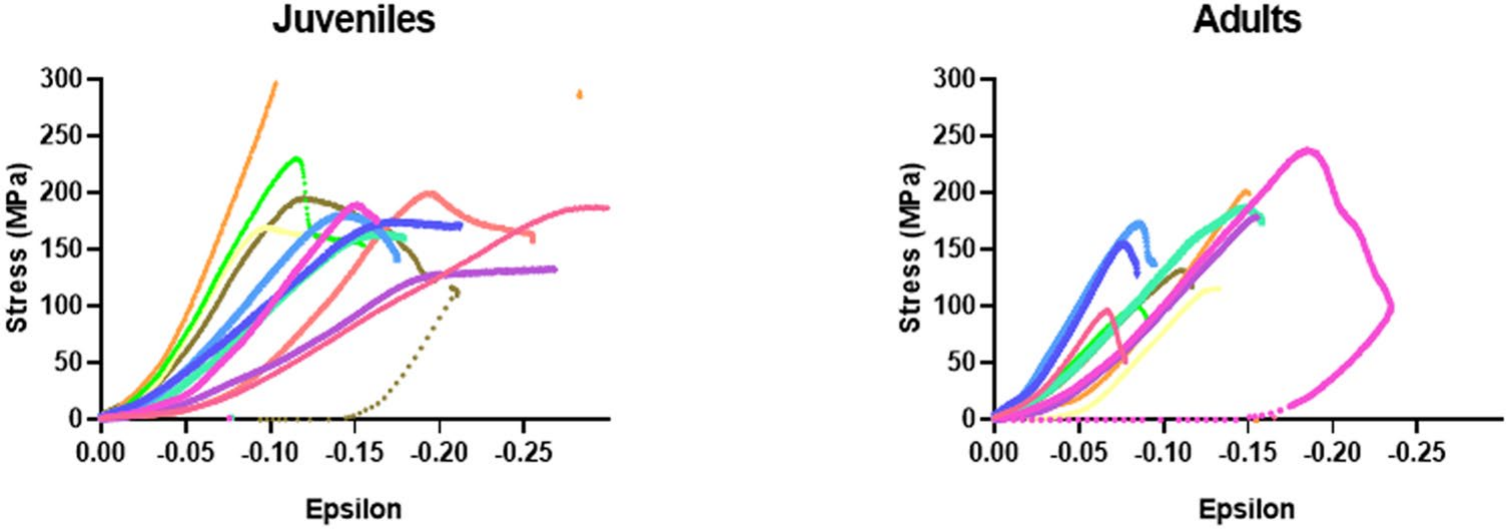
Mechanical raw data from juveniles (left) and adults (right) samples.

Results obtained for stiffness coefficients were also similar, with a trend to transverse isotropy for both children and adults bone samples (C_33_ > C_11_ = C_22_ and C_44_ = C_55_ > C_66_). All diagonal elastic coefficients were higher in adult bone samples compared to juvenile bone samples, except for C_11_ and C_22_ (not reach significance level). Concerning the compression experiments, raw curves are shown in Fig. 3. They show two trends: either a fragile or a plastic-like response. The fragile response concerns mostly the adult samples, while the plastic-like response (rather ductile) concerns mostly the juvenile ones (Fig. 3). Juveniles’ bones showed a significant higher maximal stress compared to adult bone, but with an identical elastic modulus. These results have to be looked in parallel with biochemistry measurement, where immature cross-links (DHLNL and HLNL) are significantly higher and mature cross-links are slightly lower in juvenile group compared to adults. These results lead to a ratio of immature to mature cross-links (CX) that decreases dramatically with age.

**Table 2:** Spearman correlation coefficients (r′) obtained between chronological age, elastic coefficients, elastic modus and ration of immature/mature cross-links, and the different parameters of the microstructure analysis obtained by μCT (*p < 0.05, #*p* < 0.01, Juv. represents the juvenile group).

	Age		C11		C22		C33		C44		C55		C66		E		CX	
	Juv	Adults	Juv	Adults	Juv	Adults	Juv	Adults	Juv	Adults	Juv	Adults	Juv	Adults	Juv	Adults	Juv	Adults
Age	**–**	−	**.765**^#^	− **.44bl**	**.771**^#^	− .402	.584*	− **.648**^#^	.335	− .581*	.402	− .056	.609*	− .358	.612*	− .595*	− .226	− .272
Po.V/TV	− **.528bl**	.452	− .445	− .439	− .363	− .636*	− .648*	− **.46bl**	− .429	− .432	− .424	− .057	− .402	.043	− **.797**^#^	− .445	.596*	.032
Po.S/Po.V	.394	− .159	.374	.193	.301	.486	.635*	.082	.398	.168	.385	− .071	.354	− .182	**.790**^#^	.309	− .587*	− .214
Po.N	− .567*	**.445bl**	− .56*	.054	− .653*	− .243	− **.811**^#^	− .354	− .631*	− .268	− **.675**^#^	.004	− **.679**^#^	.111	− **.734**^#^	.218	**.49bl**	.061
Po.Dm	− .265	− .045	− .121	− .250	.029	− .532*	− .314	− .114	− .116	− .171	− .108	.050	− .011	.168	− .594*	− .327	.455	.211
Po.Dm.SD	− .204	.064	− .016	− .275	.169	− .593*	− .156	− .175	− .002	− .175	.051	.164	.077	.186	− **.55bl**	− .336	.437	.125
Po.Sp	**.528bl**	− **.685**^#^	.451	.418	.248	**.457bl**	.464	.629*	.160	**.489bl**	.235	.018	.218	.207	**.51bl**	.182	− .275	.079
Po.Sp.SD	**.687**^#^	− **.742**^#^	.637*	.254	.345	.368	.266	.582*	.029	**.443bl**	.015	− .039	.213	.182	.105	.273	− .147	− .059
Conn.Den	− .109	.297	− .225	− .404	− .266	− .407	− .349	− .304	− .393	− .136	− .468bl	.243	− .231	− .243	− .371	.236	.345	.064
DA	.293	− .193	.483	.382*	.198	**.554bl**	.374	.193	.445	.021	.335	− .171	.352	.161	.594*	.191	− .599*	.089
Po.Pf	**.501bl**	− .247	.452	.132	.410	.514	**.729**^#^	.294	.468	.186	.473	− .050	.630*	− .175	**.708**^#^	.264	− .451	.025
SMI	.237	− **.479bl**	.324	− .136	.538*	− .389	**.477bl**	.143	.468bl	− .036	**.495bl**	− .004	.455	.157	− .119	− .091	.033	.214
TMD	.358	− .106	.396	**.502bl**	**.521bl**	.545*	.631*	.372	.560*	.341	.565*	− .073	.451	.007	.573*	.169	− .534*	.111
E	.612*	− .595*	.300	**.566bl**	.664*	.301	**.764**^#^	.336	.718*	.406	**.809**^#^	.371	.682*	.189		−	− **.56bl**	− .266
CX	− .226	− .272	− .390	− .047	− .467	.035	− **.687**^#^	.197	− **.819**^#^	.115	− .670*	− .044	− .621*	− .176	− **.555bl**	− .266		

Bold correspond to significant correlation.

Juv., Juveniles.

*p < 0.05, bl: borderline: 0.051 < *p* < 0.099.

^#^*p* < 0.01.

### Comparison between parameters in juveniles and adults

Table 2 reports the Spearman’s correlation coefficients obtained between the diagonal stiffness coefficients, the compression modulus E, the ratio CX and the pore network microstructure parameters. As previously published, for the juvenile bone samples, the C_33_ (axial traction compression) was negatively correlated with Po.V/TV and Po.N (r′ *resp*. − 0.65 to − 0.81) and positively correlated with Po.S/Po.V and Po.Pf (r′ *resp*. 0.64–0.73). Moreover, C_33_ was positively correlated with TMD (r′ = 0.63) and with E (r′ = 0.76) and negatively correlated to CX (r′ = −0.69). Not similar results were obtained with C_66_ (transverse shear), with only negative correlation with Po.N (r′ = −0.68, *p* = 0.008) and positively Po.Pf (r′ = 0.63). C_66_ was also correlated positively to E (r′ = 0.68) and negatively to CX (r′ = −0.62). In the adult group, C_33_ was negatively correlated with Po.V/TV (r′ = −0.65) and positively with Po.Sp and Po.Sp.SD (*resp.* 0.63 and 0.58), but none with E and CX. For the C_66_, no correlation was observed either with the microstructure of the pore network or with E and CX.

When we look at the cross-links CX, they are linked with other parameters only in the juvenile group. For example, CX is positively correlated to Po.V/TV (r′ = 0.60), and negatively correlated to Po.S/Po.V, DA, TMD, C_33_, C_44_ C_55,_ and C_66_ (*resp*. − 0.59, − 0.60, − 0.53, − 0.69, − 0.82, − 0.67, − 0.62) and borderline with E (− 0.555, *p* = 0.077).

Other stiffness coefficients show some trends with architectural parameter in adults (as noted “bl” in Table 2) but more sample need to be added to confirm those trends.

However, from a mechanical point of view, compression modulus E is highly positively correlated with age, Po.S/Po.V, DA, and Po.Pf (*resp.* 0.61, 0.79, 0.59, 0.71) and negatively correlated with Po.V/TV, Po.N, Po.Dm, and borderline with CX (*resp.* − 0.80, − 0.73, − 0.60, − 0.56), in the juvenile group. None of these results appears in adult group.

## Discussion

The purpose of this study was to add complementary information to those already published by our group on bone properties. As mentioned, we performed two supplementary measurements: a mechanical test on cubic samples already tested by ultrasounds and imaged with µCT scanner and a cross-link measurement on the surrounding bone.

Ultrasonic measurements are made at 1 or 5 MHz which corresponds to a millimetric – wavelength larger than the size of the vascular pores. It means that the ultrasonic wave interacts with an equivalent medium with homogenized properties including the effect of the microstructure (pores size, pore orientation etc.).

In term of pore network microstructure, we obtained similar conclusions than previously reported^19–21^. Some results may show discrepancy in statistics due to the standard accuracy of such measurement. However, mains results allowed the identical conclusions than the previous ones^19–21^.

**Figure 4:**
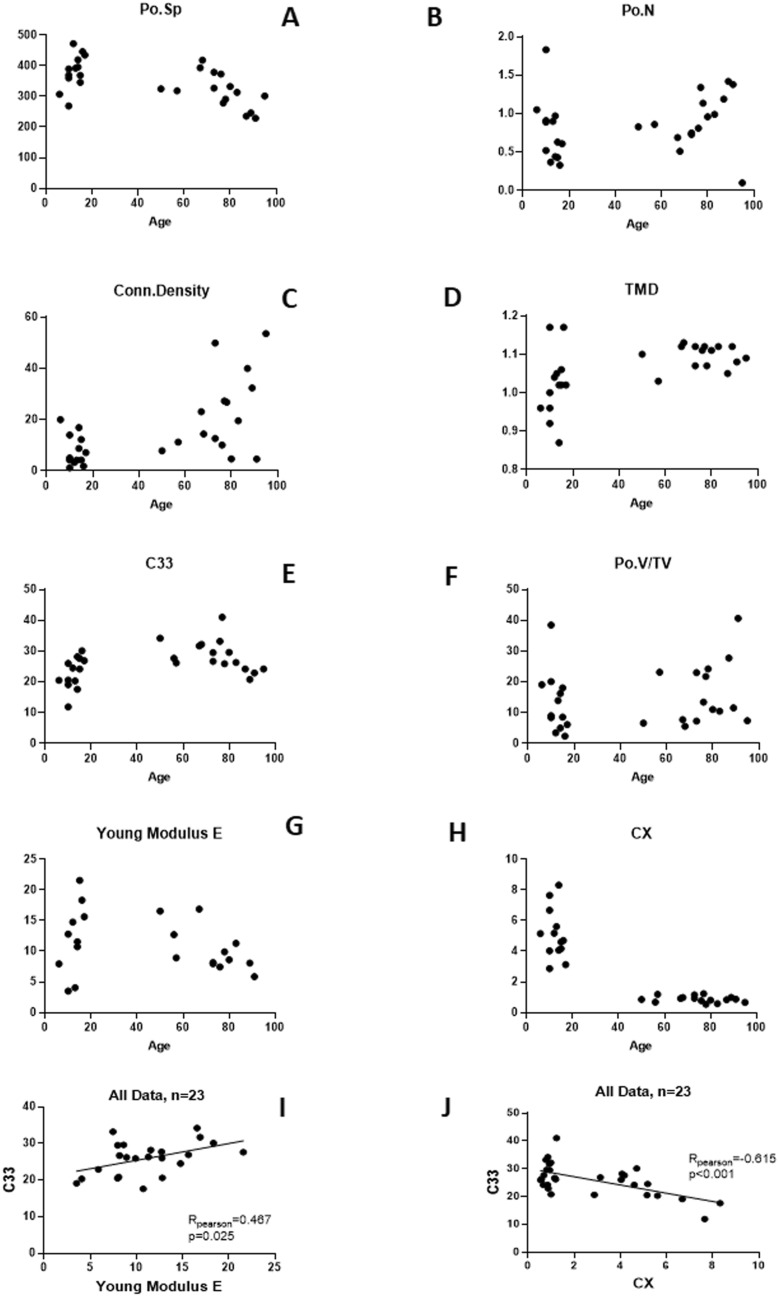
Graphs depending on age of (**A**) porosity separation Po.Sp (µm), (**B**) porosity number Po.N (1/mm), (**C**) connectivity density Conn.Density (1/mm^3^), (**D**) tissue mineral density TMD (g/cm^3^), (**E**) traction–compression stiffness coefficient C_33_ (GPa), (**F**) pore volume over tissue volume Po.V/TV (%) (**G**) compression modulus E (GPa), (**H**) cross-links ratio immature over mature CX (mmol/mol collagen). Relationships for all data between parameters traction–compression stiffness coefficient C_33_ versus, (**I**) compression modulus E (GPa), and (**J**) cross-links ratio immature over mature CX (mmol/mol collagen).

Scatterplots (Fig. 4) show the huge dispersion we can observe on different parameters during the growth and advanced age emphasizing the need to collect more samples to provide stronger findings. As Zimmermann et al. for younger cases^22^, we also observed an increasing modulus with age.

If we compare adult and children fibulae, it seems obvious that the diagonal stiffness coefficients C_ii_ (i = 1.0.6) are more representative of both microstructure parameters and CX in children bone than in adult bone (Table 2). Indeed, 8 microstructure parameters (Po.Sp.SD, Po.N, SMI, PoV/TV, PoS/PoV, Po.Pf, TMD, Po.Pf and CX) are correlated with at least one of the 6 Cii for juvenile group. And, 7 microstructure parameters (DA, PoV/TV, Po.Dm, Po.Dm.SD, TMD, Po.SP, Po.Sp.SD) are correlated with only the 3 first Cii (related to compression waves—compression/dilatation) for adult group.

It means that the ultrasonic measurement can account for several determinants of the children bone and be a relevant modality to assess children bone quality. In children bone, the C_33_ is the diagonal stiffness coefficient that is the most sensitive to both microstructure parameters and CX. And yet, it is the most accessible C_ii_ by axial transmission measurement^29^ which opens promising prospects in terms of clinical implementation for paediatrics.

It is noteworthy that E is also representative of both microstructure parameters and CX only for juvenile bone fibula and not at all for adult bone fibula. This is coherent with the fact that E represents also a stiffness (despite being measured differently). Nevertheless, the compression test is destructive and not clinically feasible. Another important mechanical difference is that children bones exhibit plastic-like behaviour while adult ones are fragile. Berteau et al. found in 3-point bending similar results^9^ showing that a collagen matrix with more immature cross-links (i.e. a higher immature/mature cross-link ratio) is more likely to plastically deform before fracture. Further investigation of this plastic-response will be important for bone with pathological weakness as osteogenesis imperfecta.

Ultrasounds measurements and compression test provide mechanical characteristics of the fibulae at mesoscopic scale. Both seem to be less representative of the structural and biochemical parameters as bone ages. Maybe this trend is specific to fibula which is not a weight-bearing bone and could explain that the mechanical environment (external forces) is less determinant in the evolution of this bone with aging especially in the microstructure orientation so the correlation between microstructural parameters and mesoscopic stiffness coefficients is lost with age. This assumption is in accordance with a recent study on the skull^30^. Several papers demonstrate that in femur or tibia for example which are bearing bones, mesoscopic mechanics are highly correlated to microstructure even for adult cortical bone^31–34^. This analysis leads to conclude that ultrasounds can be a more responsive tool to assess cortical bone quality in child (growing) bone than in ageing bone at anatomical sites such as radius, skull or fibula (non-weight bearing bone).

### Limitations

The value of E obtained by compression test is lower than expected (of 15–20 GPa). This can be explained by the fact that we do not measure the Young’s modulus of the bone specimen but an apparent modulus because the deformation in the linear portion of the stress–strain curve is not purely elastic^35^.

Moreover, decrease of the apparent modulus may come from the sample shape. First, the samples are cubic while it would be better to have elongated samples in the direction of loading to get rid of boundary effects (contact with the machine heads). Second, despite our care, the samples are not perfectly cubic, which impact the relation between stress and strain, as we measure the strain only on one surface.

Despite those limitations, this is suggesting that ultrasounds measurements could predict elastic coefficients, but also bone mineral density, microstructure properties and the ratio between immature/mature cross-links. This is of great interest for the future in a long-term perspective, for children in a hospital environment, in which such measure is not invasive, not stressful, do not necessity an injection, and so forth. First promising steps have been done in a clinical trial by French colleagues^36–38^.

## Conclusion

This study shows trends saying that ultrasounds measurements could predict both the mechanical and microstructural properties, but also give information on the ratio of immature/mature cross-linking, at least on juvenile bone. To further confirm these observations, more samples will be needed to have a better statistical validation.

## Supplementary information

Supplementary Information.
