# Supplementary material for: Ultrasounds could be considered as a future tool for probing growing bone properties
Source: Sci Rep. 2020 Sep 24;10:15698. doi: 10.1038/s41598-020-72776-z (PMC7518273; doi:10.1038/s41598-020-72776-z)
Supplement: Supplementary file 1 — Supplementary Information. [file 41598_2020_72776_MOESM1_ESM.docx]

Title**:**

“Ultrasounds could be considered as a future tool for probing growing bone properties”

Emmanuelle LEFEVRE, PhD^1, 2^ **+**

Cécile BARON, PhD ^1, 2,^ **+**

Evelyne GINEYTS, PhD ^3^

Yohann BALA, PhD ^3, 6^

Hakim GHARBI, ^4^

Jean-Marc ALLAIN, PhD ^4, 5^

Philippe LASAYGUES, PhD ^7^

Martine PITHIOUX, PhD ^1, 2^

Hélène FOLLET, PhD ^3^*

**Supplementary Table S1** – Juvenile Femurs – raw data

| **Age** | **Sex** | **C11** | **C22** | **C33** | **C44** | **C55** | **C66** | **DPD** | **PYD** | **DHLNL** | **HLNL** | **[DHLNL+ HLNL]** | **[PYD+ DPD]** | **[PYD/ DPD]** | **CX** |
| --- | --- | --- | --- | --- | --- | --- | --- | --- | --- | --- | --- | --- | --- | --- | --- |
| 1 | M | 93 | 96 | 131 | 29 | 26 | 26 | 44 | 337 | 3017 | 718 | 3735 | 381 | 7.58 | 9.80 |
| 15 | F | 131 | 183 | 287 | 50 | 51 | 40 | 60 | 449 | 1962 | 700 | 2662 | 509 | 7.50 | 5.23 |
| 10 | M |  |  |  |  |  |  | 53 | 309 | 3010 | 731 | 3742 | 362 | 5.80 | 10.33 |
| 5 | F | 15 | 13 | 24 | 3 | 3 | 3 | 66 | 431 | 2199 | 729 | 2929 | 498 | 6.51 | 5.89 |
| 1 | F | 133 | 134 | 169 | 37 | 31 | 23 | 54 | 343 | 2955 | 658 | 3613 | 397 | 6.35 | 9.10 |
| 1 | F | 86 | 93 | 140 | 25 | 22 | 21 | 55 | 478 | 2630 | 604 | 3234 | 533 | 8.75 | 6.07 |
| 1 | F | 142 | 153 | 184 | 42 | 35 | 29 | 83 | 427 | 3155 | 885 | 4040 | 511 | 5.13 | 7.91 |
| 1 | F | 121 | 119 | 182 | 33 | 32 | 24 | 69 | 455 | 3014 | 658 | 3672 | 524 | 6.64 | 7.01 |
|  | **Mean** | 103 | 113 | 160 | 31 | 29 | 24 | 61 | 404 | 2743 | 710 | 3453 | 464 | 6.78 | 7.67 |
|  | (±SD) | 44 | 54 | 78 | 15 | 14 | 11 | 12 | 64 | 440 | 83 | 467 | 71 | 1.13 | 1.92 |

**With CX=[DHLNL+ HLNL]/ [PYD + DPD]**
